# Population-weighted degree-days: The global shift between heating and cooling

**DOI:** 10.1016/j.enbuild.2022.112315

**Published:** 2022-09-15

**Authors:** H. Kennard, T. Oreszczyn, M. Mistry, I. Hamilton

**Affiliations:** a Energy Institute, The Bartlett School of Environment, Energy and Resources, UCL, London, UK; b Department of Economics, Ca’ Foscari University of Venice, Venice, Italy; c Department of Public Health, Environments and Society, London School of Hygiene & Tropical Medicine, London, UK

**Keywords:** Degree-days, Heating demand, Cooling demand, Climate change, Global Warming, Population

## Abstract

Anthropogenic greenhouse gas emissions are driving global increases in temperature. This rise will likely lead to an increase in demand for cooling in the coming years. However, increasing temperatures are not the main explanatory factor for why the world is moving towards more cooling. This paper compares population and area-weighted cooling and heating degree-days derived using ERA5-Land reanalysis temperature, to show that population growth in warmer parts of the world drives cooling demand globally. The analysis shows that mean global area-weighted heating degree-days have fallen 8.46 °C days/year, whereas population-weighted heating degree-days have fallen by 12.5 °C days/year. At the same time, mean global area-weighted cooling degree-days have risen by 3.0 °C days/year, while population-weighted cooling degree-days have risen at 6.0 °C days/year. By using sub-country analysis, this paper shows that population-weighted degree-days can substantially differ from area-weighted degree-days. Finally, the findings highlight that the choice of heating and cooling degree-day base temperature is the most important parameter in the variability of degree-days and will need to be understood better in order to accurately account for future heating and cooling energy demand.

## Highlights

• Globally, population weighting substantially changes degree-days rate of change.
• Choice of base temperature strongly influences degree-day calculation.
• For typical base temperatures, heating degree-days are falling at twice the rate that cooling degree days are increasing.
• Within countries, heterogeneity in population changes determines relative changes in heating and cooling degree-days.

## 1 Introduction

Analysis by the International Energy Agency (IEA) shows that in 2019, space heating in buildings accounted for 12% of global energy use and 13% (4.3 Gt) of CO_2_ emissions. Space cooling accounted for a much smaller proportion, at 2% of global energy and 3% (1 Gt) of CO_2_ emissions [1]. Since at least 1928, degree-days have been used to explain how this energy demand changes with weather and climate [2], with both building and national energy demands often normalised by locally measured degree-days using a nationally defined base temperature. This paper examines how, over the last four decades, the location and relative growth of the global population combined with climate change are shifting degree-days from heating-dominated to cooling-dominated.

Degree-day calculations sum the difference between the average daily temperature and a reference base temperature across the year; an example is the widely used American Society of Heating, Refrigerating, and Air-Conditioning (ASHRAE) method [3], [4], [5]. For heating degree-days (HDDs), deviations *below* the base temperature are included whereas cooling degree-days (CDDs) include deviations *above* the base temperature (see methods). The base temperature is critical to the calculation of degree-days and different countries have used different standard or reference base temperatures. The heterogeneity in base temperatures used by different studies has received attention. Using a case study examining Birmingham UK, Azevedo et al. [6] argued for the use of a universal base temperature based on the average external temperature of the region under examination. As part of their analysis, the authors conducted a review of base temperatures used in the degree-days literature, finding substantial heterogeneity of base temperatures used, even within the same country. Across the literature they examined, the mean population-weighted base temperatures was T_base_^HDD^ = 18.1 °C and T_base_^CDD^ = 22.0 °C.

Justifications for a particular base temperature choice can be made by noting that base temperature is not a fixed parameter, but one that changes with internal temperature for the thermal comfort of the building occupants, building design and internal heat gains [6]). Theoretically the temperature at which the heating or cooling system turns on is called the balance temperature [7] which is synonymous with the base temperature; this is a function of the internal demand temperature, the building heat loss and the incidental heat gains from appliances, hot water, people, and the sun. A study by Meng et al. [8] illustrates that the correct choice of base temperature is essential for accurate buildings energy use prediction, and that the use of multiple heat fluxes though a building, such as incident solar radiation and convective loses, improve the accuracy of energy use predictions.

Heating and cooling seasons have also been defined in some analyses e.g. the Intergovernmental Panel on Climate Change (IPCC) Sixth Assessment Report (AR6) [9] calculate HDDs and CDDs during a defined heating/cooling season using work by Spinoni et al. [10], for which the heating season runs October 1st to March 31st and in our analysis we have not taken this approach. Historically this approach may have been valued where a significant effort was required to turn on or off the heating or cooling system, and may still apply to some types of heating or cooling, for example district heating systems. However, with the advent of smart controls, many heating and cooling systems are permanently on but controlled by temperature sensors in the building.

A study by Harvey [11] argued that three reference temperatures were appropriate in developing HDD and CDDs with respect to buildings energy use, namely the indoor thermostat setting, the external temperature at which internal heat gains from people, equipment and lighting equal heat losses and finally, the external temperature at which solar gains alone match heat loss. However, there is a paucity of information regarding internal temperatures and internal gains at a global scale.

The impacts on mortality of exposure to ambient temperatures have been well described using epidemiological methods [12], where evidence suggests the optimal ambient temperature differs by location [13]. The characteristics of the built environment mediate and modify a population’s exposure to ambient temperatures [14], [15], with the relative balance between HDDs and CDDs determining the type of heating/cooling system installed. In turn, this drives national energy use and greenhouse gas emissions.

This study looks at the change in population-weighted degree-days between 1981 and 2018 by weighting the local degree-day calculation using local population counts before a country-wide aggregation is performed. An early use of population weighting for degree-days was by Taylor [2] in a study of Canada, which demonstrated the need for population weighting in countries where populations are concentrated in areas which are not representative of the mean degree-days of the region or country as a whole. A study by Guttman [16] of the lower 48 states of the USA also used population weighting, and found that during the 1970s shifts in population distribution reduced heating demand but increased cooling demand.

Tol [17] uses population weighting to derive the average global temperature experienced by the human population throughout the 20th century. Alongside showing the impact of global warming, the study shows that the urban heat island effect substantially increases the amount of experienced warming, and that international migration has had a minor negative impact on the average. A study of exposure to extreme heat in urban settings found that two-thirds of the annual increase in total exposure-days between 1983 and 2016 was accounted for by population growth, with the remaining third explained by increasing temperatures [18]. These studies underscore the importance of understanding inhomogeneities in population changes both spatially and temporally.

Atalla, Gualdi and Lanza [19] produced a population-weighted degree-days dataset for 147 countries between 1948 and 2013. The base temperatures used for the 2 m height temperature index, for both CDDs and HDDs, were 15.6 °C, 18.3 °C and 21.1 °C (60°F, 65°F and 70°F respectively). Spinoni et al. [20] produced projections for population-weighted degree-days under different development scenarios, finding that population weighting means the increase in CDDs outbalances the decrease in HDDs as temperatures increase, under almost all future projections. However, analysis by Deroubaix et al. [21] of projections in heating and cooling trends under climate change point to substantial uncertainties. Biardeau et al. [22] use population-weighted CDDs to rank countries and cities and highlight potential unmet cooling demand in countries such as India, China, and Indonesia, an important driver of future global emissions.

Several analyses project increasing total demand for cooling as climate change projections show higher mean global temperatures in the coming decades. Analysis from the IEA suggests an increase of global mean temperature of 1 °C by 2050 will lead to a 25% increase in CDDs [23]**.** Indeed, a strong correlation between annual mean temperature and degree-days has been shown by Mourshed [24]. The analysis of 5511 weather station locations suggests a strong correlation between mean external temperature and HDDs (and CDDs), although the relationship deviates from linearity above (below) the base temperature of 10 °C for HDDs (18.3 °C for CDDs^1^). The linear regression employed by Mourshed [24] suggests an 11% increase in CDDs for a 1 °C increase above a global average of 14 °C.

This paper contributes insights into the differences between area weighting and population weighting for degree-days calculations, both at a global level and through country level examples. It underscores how sensitive degree-days calculations are to both the choice of base temperature, as well as the weighting method. Finally, it contributes a dataset of both area-weighted and population-weighted degree-days between 1981 and 2018 at a range of base temperatures for each country.

### 1.1 Research questions

Here we address the following research questions:

1. How have area-weighted and population-weighted heating and cooling degree-days changed historically, both globally and at the country level?
2. What is the relative importance in population migration and expansion compared to climate change in determining if the global energy demand is driven by heating or cooling?
3. How important is the choice of the degree-day base temperature in this change over time?

## 2 Theory & calculation

**Fig. 1:**
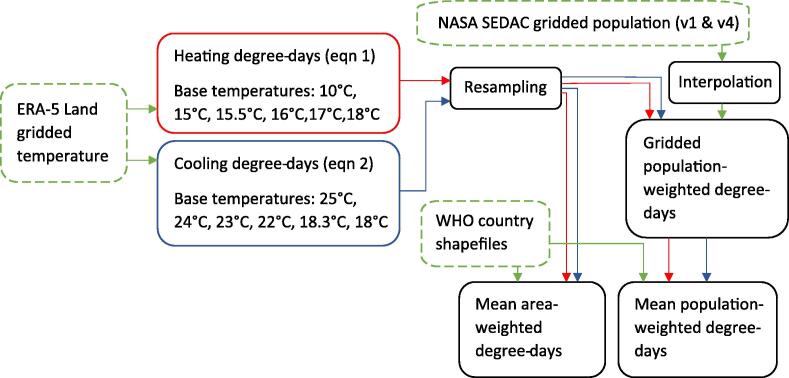
Method flowchart summarising process to by which the input data sources (green dotted outline) produce the resultant dataset.

The main object of analysis used in this study is gridded annual degree-day and population rasters. A raster is gridded image corresponding to a given geographic projection of the earth’s surface where each grid-cell is ascribed a number. As such total populations (or degree-days) may be calculated by summing over the area in question. In this case, the population-weighted degree-day was produced by multiplying the value of the degree-day in a given cell with the population in the same cell, summing over the area, and the dividing by the total population in that area. The country areas were provided by a WHO shapefile mask. The complete data processing path is summarised in Fig. 1.

The format used in this study are both .nc4 and .tiff (under the GeoTIFF standard). The years 1981 to 2018 inclusive were considered in this study. The area-weighted estimates of degree-days by country were calculated by summing over the area falling inside the WHO shapefile mask for each country.

Annual gridded degree-days at 0.1 × 0.1 deg (∼10 × 10 km at the equator) spatial resolution were computed using the ERA5-Land temperatures [25] at the following base temperatures: HDD (10 °C, 15 °C, 15.5 °C, 16 °C,17 °C,18 °C) and CDD (25 °C, 24 °C, 23 °C, 22 °C, 18.3 °C, 18 °C). For simplicity, HDD at base temperature of 18 °C is denoted HDD_18_, with the same convention used for other base temperatures. The base temperatures used in this study were chosen to coincide with the typical base temperatures used in different geographic locations (see Azevedo et al. ^6^).

Briefly, CDD and HDD (Units **°C days)** were calculated following ASHRAE [3] and Mistry [4], defined as follows:

(1) $$\mathrm{CDD}=\sum_{i=1}^{n}{\left(T_{d}-T_{b}\right)}^{+}$$

(2) $$\mathrm{HDD}=\sum_{i=1}^{n}{\left(T_{b}-T_{d}\right)}^{+}$$

where ‘+’ signifies only positive values accumulate over n days in the chosen time period (here years). T_d_ and T_b_ in equations 1–2 represent the daily mean outdoor air and base temperatures respectively.

The global gridded T_d_ (°C) in our study is assembled using the near-surface hourly temperature of ERA5-Land climate reanalysis [24], the most recent iteration of global reanalysis products from the European Centre for Medium Range Weather Forecasts (ECMWF) Copernicus programme that provides land-surface data at ∼10 km resolution.

Gridded population estimates for each year were generated using the combination of two datasets published by NASA’s Socioeconomic Data and Applications Center (SEDAC). For the years 1981 to 2000 “Global Population Count Grid Time Series Estimates, v1” was used [26] and for 2001 to 2018 “Gridded Population of the World (GPW), v4” [27]. Each dataset provides population count estimates at 5-year intervals, the intervening years were therefore interpolated to provide the same time-resolution as the degree-day data. For both population datasets the resolution is 30 sec (0.008333 × 0.008333 deg) which is approximately 1 km at the equator. The degree-day rasters were rescaled to the 30 sec resolution in order to multiply them with the population rasters.

For each country in the WHO shapefile dataset, the process is repeated using a shapefile which masks the population and degree-day TIFFs. For each country the area-weighted degree-days for each base temperature was also calculated.

For the country illustrations given below the graphed population changes are shown between the years 2005 and 2014 as these correspond the survey years which the UN population estimates are based on.

## 3 Results

### 3.1 Global picture

**Fig. 2:**
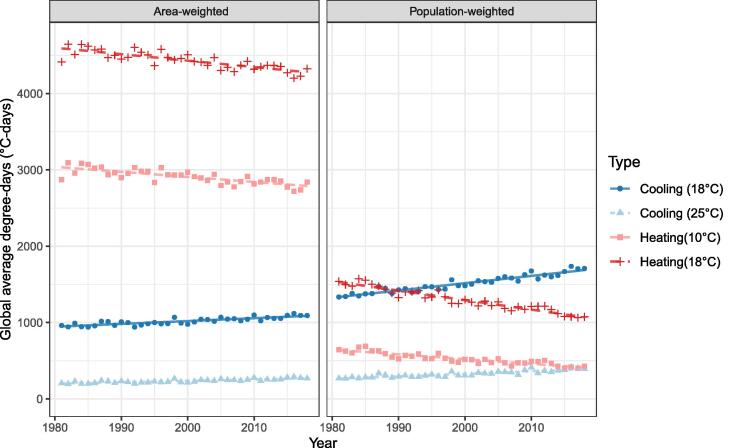
Comparison between area-weighted and population-weighted global degree-days by year at given base temperatures.

Fig. 2 shows the change over time of total degree-days by base temperature (using the range from the literature) and type (cooling or heating). Other intermediate base temperature values are given below in Table 1. Under both weighting types, CDDs have increased between 1981 and 2018 and HDDs have decreased, which is consistent with observed increases in global mean surface temperature.

**Table 1:** Linear Regression of cooling and heating degree-days from 1981 to 2018 for different base temperatures and weighting. Gradient (m) and intersection (c) of equation DD = m × Year + c, with R^2^ and 95% confidence intervals.

	Area			Population		
DD type	R^2^	m	c	R^2^	m	c
CDD 18	0.72	3.76 ± 0.88	−6496 ± 1753	0.90	9.55 ± 1.18	−17591 ± 2366
CDD 18.3	0.64	4.03 ± 1.13	−7087 ± 2262	0.90	9.41 ± 1.20	−17379 ± 2406
CDD 22	0.67	2.96 ± 0.79	−5407 ± 1573	0.85	6.00 ± 0.96	−11261 ± 1916
CDD 23	0.68	2.50 ± 0.64	−4595 ± 1289	0.83	5.04 ± 0.87	−9495 ± 1741
CDD 24	0.68	2.31 ± 0.60	−4316 ± 1204	0.80	4.21 ± 0.80	−7976 ± 1607
CDD 25	0.68	1.84 ± 0.48	−3440 ± 951	0.76	3.31 ± 0.70	−6284 ± 1405
HDD 10	0.61	−6.61 ± 2.01	16134 ± 4025	0.82	−6.20 ± 1.11	12919 ± 2211
HDD 15	0.66	−7.76 ± 2.12	19332 ± 4233	0.89	−9.85 ± 1.33	20634 ± 2651
HDD 15.5	0.66	−7.88 ± 2.13	19660 ± 4261	0.89	−10.27 ± 1.35	21527 ± 2689
HDD 16	0.66	−7.99 ± 2.15	19992 ± 4289	0.90	−10.70 ± 1.36	22438 ± 2728
HDD 17	0.67	−8.23 ± 2.18	20667 ± 4349	0.91	−11.57 ± 1.40	24303 ± 2809
HDD 18	0.68	−8.46 ± 2.21	21356 ± 4413	0.91	−12.46 ± 1.45	26215 ± 2894

Table 1 shows the rate of change (m) of area-weighted DDs and population-weighted DDs, for the given base-temperatures. For area-weighted DDs, the rate of reduction of HDDs is greater than the rate of increase of CDDs; for the typical base temperatures derived from Azevedo et al. [6] (see section 1) HDD_18_ is falling at 2.8x the rate that CDD_22_ is increasing. If the global warming trend established over the past four decades continues, heating would be expected to fall faster than CDDs increase, implying the potential for a net reduction in buildings energy use. However, importantly, this effect is offset when considering population-weighted degree-days, for which HDD_18_ is falling at 2.0x the rate that CDD_22_ is increasing, and for particular combinations of base temperatures, cooling degree-days are growing faster than heating degree-days are falling. This implies that population increases in areas which have relatively high cooling demands offset reductions in heating demand as the planet warms.

Again taking typical values for the base-temperature of 18 **°**C for heating and 22 **°**C for cooling, using the given regressions in Table 1, a year can be inferred at which CDDs and HDDs have equal value. For area weighting this is 2344, and for population weighting it is 2030. Other values for alternative combinations of base-temperatures are given in Table 2. The observation that the year for which this occurs is much earlier for population weighting than area weighting further illustrates the impact of population growth in regions of relatively higher CDDs.

**Table 2:** Implied year where CDDs and HDDs have the same value, by weighting type and base temperature. Intercept years which fall in the range of the available data are given in bold. For these, the confidence interval is on the order of ± 3 years (see SI for further information).

		Heating Degree-days					
Area-weighted	Base temperature	10 **°**C	15 **°**C	15.5 **°**C	16 **°**C	17 **°**C	18 **°**C
Cooling Degree-days	18 **°**C	2182	2242	2247	2254	2265	2279
	18.3 **°**C	2182	2241	2246	2253	2264	2277
	22 **°**C	2251	2308	2312	2320	2330	2344
	23 **°**C	2275	2332	2337	2344	2354	2368
	24 **°**C	2293	2348	2353	2360	2370	2384
	25 **°**C	2316	2372	2377	2384	2394	2407

		Heating Degree-days					
Population-weighted	Base temperature	10**°**C	15**°**C	15.5**°**C	16**°**C	17**°**C	18**°**C
Cooling Degree-days	18**°**C	1937	1970	1974	1977	**1984**	**1990**
	18.3**°**C	1941	1974	1977	1980	**1987**	**1993**
	22**°**C	**1982**	**2012**	**2015**	**2018**	2024	2030
	23**°**C	**1994**	2023	2026	2029	2035	2041
	24**°**C	**2007**	2035	2037	2040	2046	2051
	25**°**C	2019	2045	2048	2050	2056	2061

**Fig. 3:**
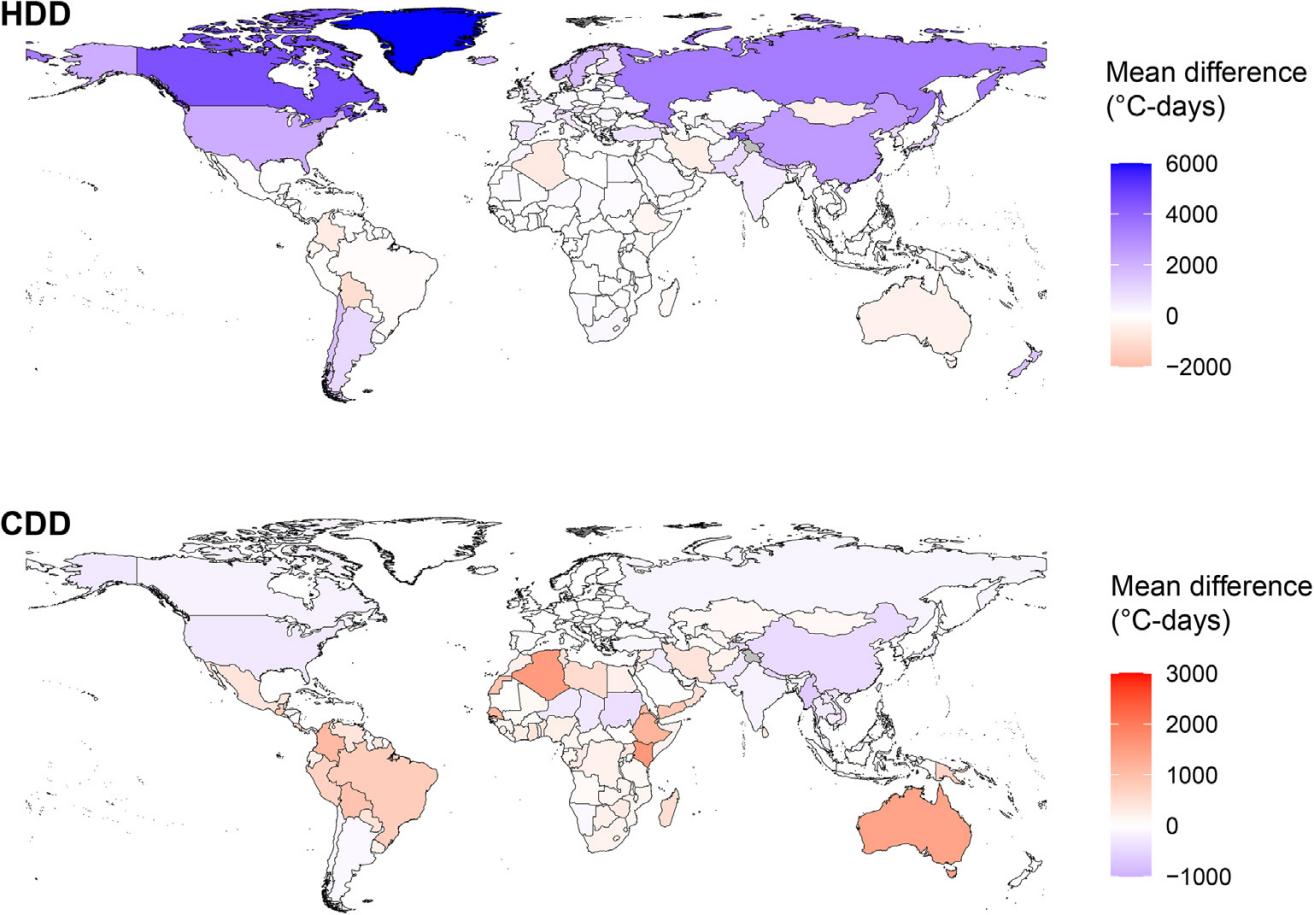
Mean difference between area-weighted population-weighted degree-days at base temperature of 18 °C, 1981 – 2018. Upper panel: heating degree-days. Lower panel: Cooling degree-day.

Fig. 3 shows the mean difference between area-weighted and population-weighted degree-days. For heating, countries such as Canada and Russia show large differences as much of their population is concentrated in regions of the country with lower heating demand. The converse is true of countries such as Australia, Brazil, and Algeria where populations are situated in coastal regions which have relatively lower CDDs.

### 3.2 Country analysis

The implications of the rate of change of HDDs and CDDs differs depending on whether population or area weighting is used. In order to demonstrate this, the following section examines three examples, Italy, Bolivia and the United Kingdom with respect to these differences. These countries were chosen as they all have similar geographic extent north to south (around 1000 km), have experienced population increases over the study period, but crucially have different cooling and heating requirements.

**Fig. 4:**
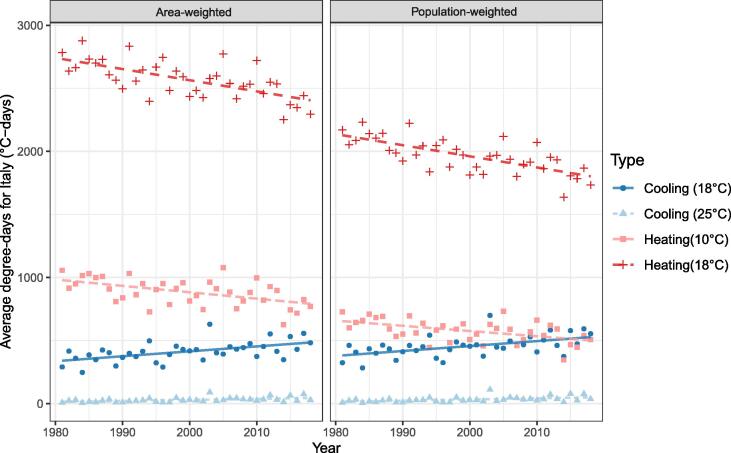
Area-weighted and population-weighted degree-days for Italy by year at given base temperatures.

**Fig. 5:**
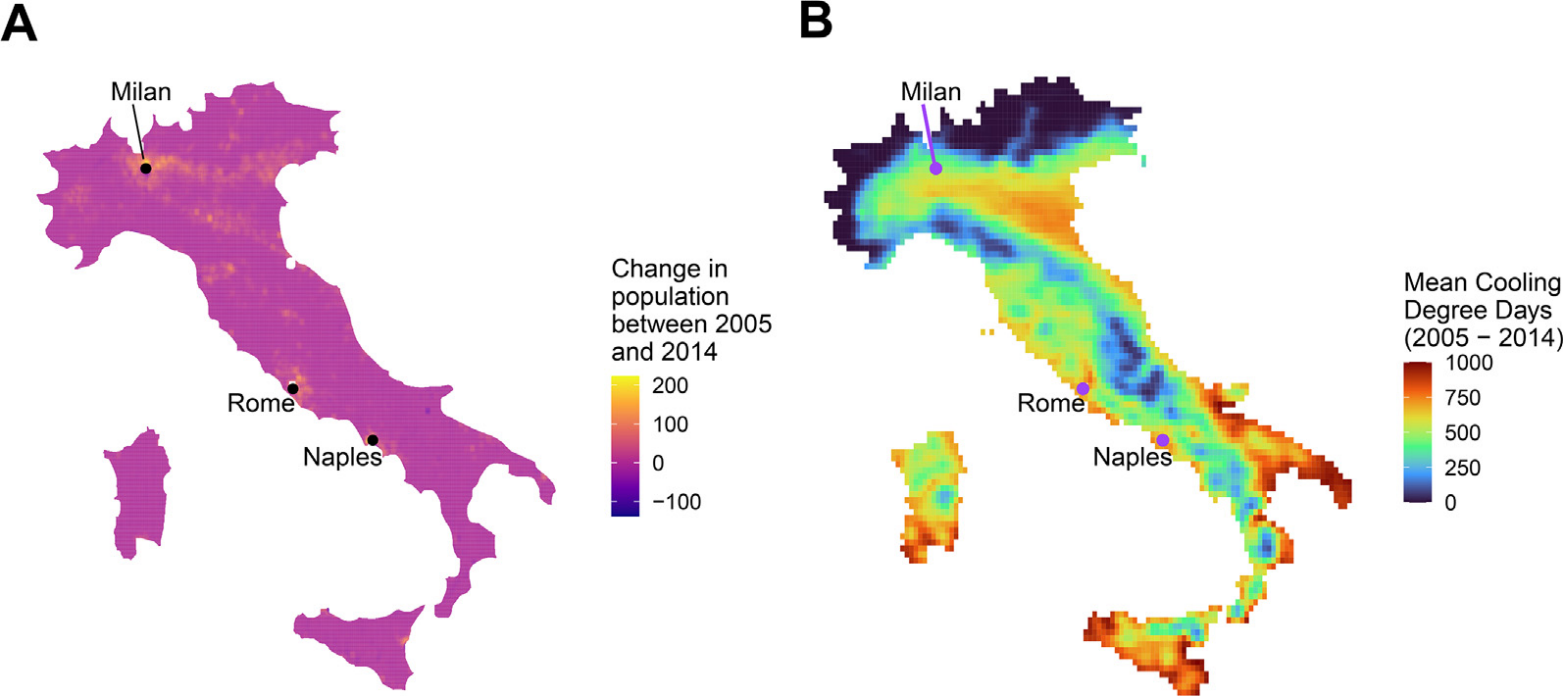
A) Population-change in Italy between 2005 and 2014 (persons/cell). Total change: 2,354,470. Mean change/cell: 4.94. B) Mean 18 °C area-weighted cooling degree-days during the same period (°C-days).

Fig. 4 shows little difference between area-weighted and population-weighted degree-days for Italy over the study period. Fig. 5 shows that despite heterogeneous population change, concentrated in the urban centres of Milan and Rome, the population has increased in locations for which the DDs are representative of the country as a whole.

**Fig. 6:**
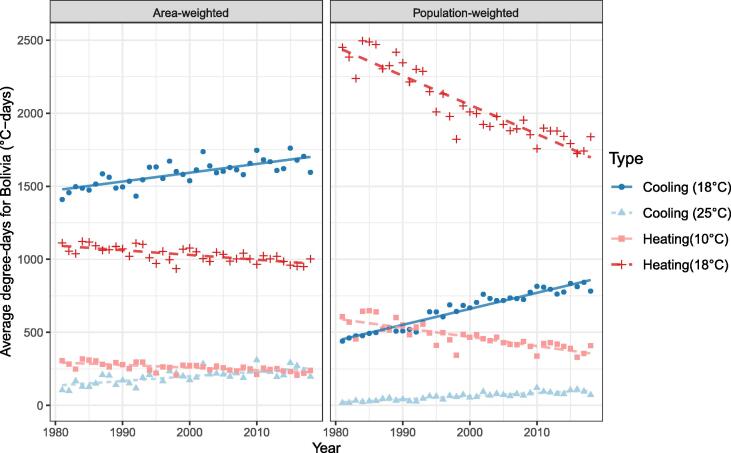
Area-weighted and population-weighted degree-days for Bolivia by year at given base temperatures.

**Fig. 7:**
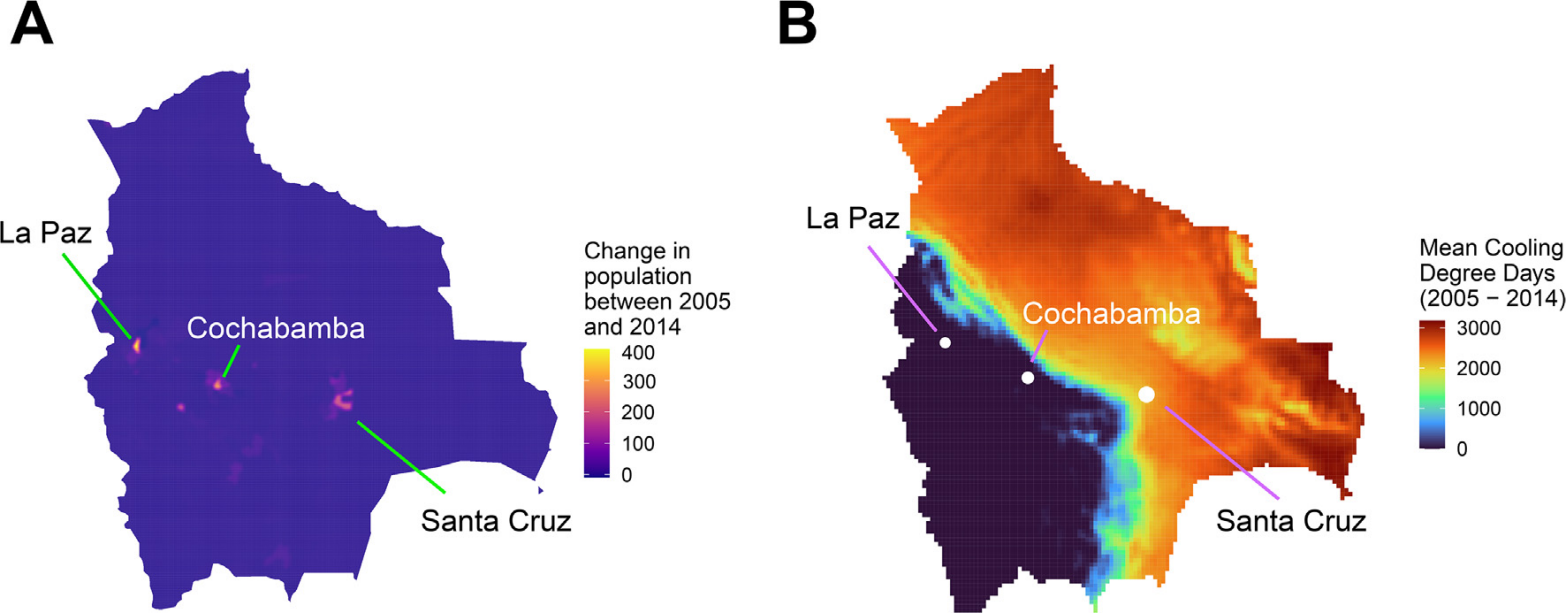
A) Population change in Bolivia between 2005 and 2014 (persons/cell). Total change: 1,624,084. Mean change/cell: 1.22. B) Mean 18 °C area-weighted cooling degree-days across the same period (°C-days).

The converse is true of Bolivia, for which the picture is very different for area and population weighting (Fig. 6, Fig. 7). Population increased mainly in La Paz, Cochabamba and Santa Cruz de la Sierra. However, the cool Andean Plateau dominates the south-eastern portion of the country and has radically different heating demands than the warm Amazon basin to the west.

**Fig. 8:**
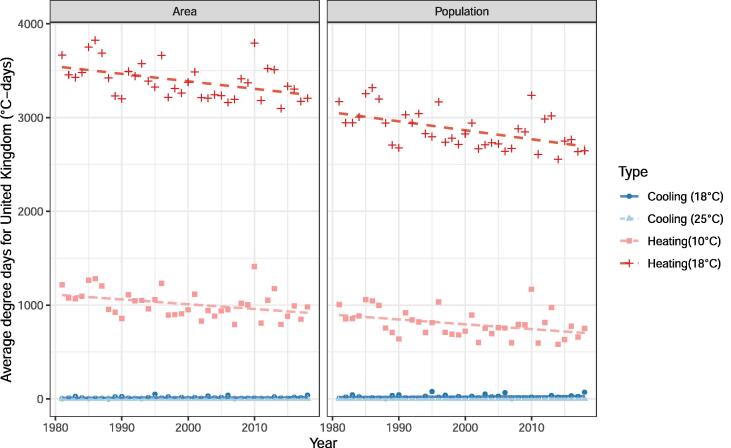
Area-weighted and population-weighted degree-days for UK by year at given base temperatures.

**Fig. 9:**
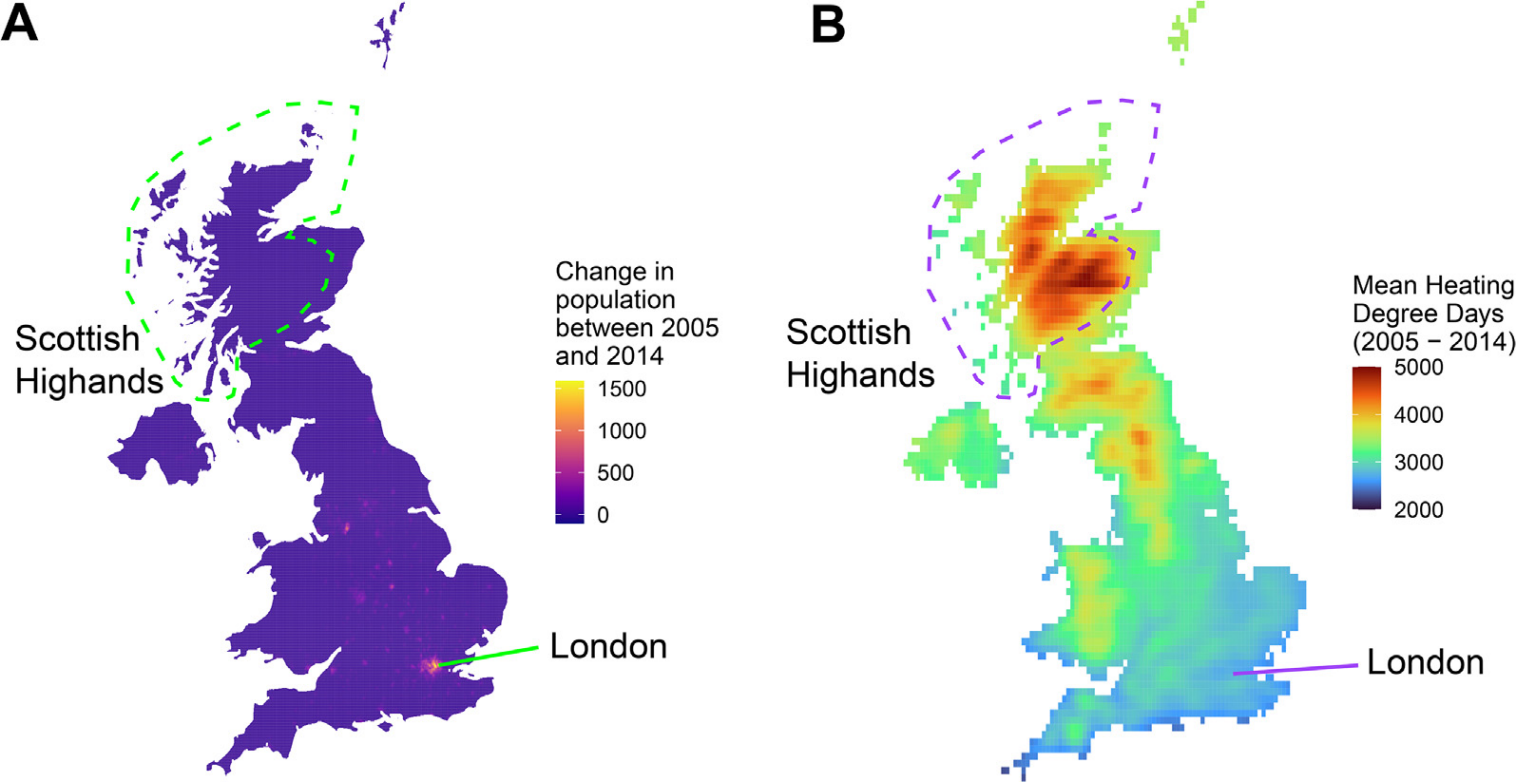
A) Population change in the United Kingdom between 2005 and 2014 (persons/cell). Total change: 4,057,868. Mean change/cell: 8.41. B) Mean 18 °C heating degree-days across the same period (°C-days).

Population has increased in the UK in an area with approximately uniform HDDs. If there had been large population increase in the Scottish Highlands (see Fig. 9), for example, there would be substantial differences in the gradients of the lines shown in Fig. 8. For countries with temperature monitoring stations located in regions which approximately correspond to population distribution then the nationally computed degree-days will equal population-weighted degree-days, this is explored in the supplementary information comparing the estimates given here with those derived by the UK government department responsible for energy (BEIS).

## 4 Discussion

This paper has demonstrated that global mean area-weighted and population-weighted degree-days have changed substantially in the past 40 years. During this period, HDDs have decreased, and CDDs have increased. The changes in area-weighted degree-days are in line with the observed global warming in our study period. Population-weighted CDDs are increasing at a faster rate than area-weighted CDDs, which is explained by increases in populations in warmer regions.

This effect occurs both globally and within countries, such as Bolivia where populations have grown in regions which have higher cooling requirements than other parts of the country. However, this study also demonstrates that degree-days calculations are heavily impacted by choice of base-temperature. Depending on the choice of base-temperature, the year at which CDDs dominate HDDs can vary from the 1930 s to the 2060 s. This level of ambiguity in basic parameters of a system demands further work for establishing in-use base temperatures from the global building stock.

However, the picture is further complicated by the observation that buildings energy use differs greatly by occupant behaviour, cultural practices, and economic constraints as well as the physical characteristics of local environments and building construction types. The parameters are not fixed and will vary substantially at decadal timescales. In this respect, the United Kingdom can be used an instructive example. Given that internal mean temperatures are known to have changed in the last 40 years [28] the base temperature for the UK housing stock will have changed over this period. However, at the same time passive heating has increased due to higher levels of insulation.

Historically it has been far easier to actively heat a building than cool it. At a global scale, rapid urbanisation and the increased use of air conditioning worldwide could lead to substantial increase in energy demand for cooling, while existing heating demand in colder regions would persist. Accurate modelling of heating and cooling demands will be essential for both the management of energy demand as the earth’s climate warms as well as the provision of healthy and comfortable internal environments. Better global data on the demand temperatures, levels of incidental gains and heat loss of buildings and in practice heating and cooling system efficiencies are required to establish how the relative impact of climate change and population location will impact global energy use. With increased global uptake of smart meters, the direct measurement of balance temperatures is now possible.

### 4.1 Limitations and further work

While the impact of humidity undoubtedly influences thermal comfort assessment, especially in warmer climates where increasing humidity leads to increased cooling requirements [29], the consideration of it here would introduce more complexity than the scope of this paper allows. For example, Heat index, Humidex and Environmental Stress Index are all used by Atalla et al. in their study of degree-days [19], consideration of each of these indices and their relationship with population weighting could form the basis of a future work.

While the statistical uncertainty of the analysis undertaken here is addressed in the results and supplementary information sections, there are additional sources of uncertainty which are not explicitly calculated here. The two main sources of uncertainty arise from the ERA5-Land input data from which degree-days are computed, and the NASA SEDAC population rasters which are used to produce the population weighting. There is likely a further uncertainty associated with edge effects of the country shapefiles which are used to define the national boundaries. While these uncertainties are not likely to be large, explicit calculation of them would constitute an interesting extension to the present study.

## 5 Conclusions

This paper has demonstrated the importance of population weighting in degree days calculations. There are statistically significant differences (at 95% confidence) between the global rate of change of population-weighted cooling degree-days and area-weighted cooling degree-days over a 38-year period (1981 – 2018) across all base temperatures considered. Depending on choice of base-temperature, population-weighted cooling degree-days have increased at twice the rate of area-weighted degree-days (1.8x – 2.5x, at 18 °C to 25 °C base temperature), suggesting population changes are driving cooling demand or need for cooling (where demand unmet).

The differences between area-weighted and population-weighted heating degree days are only statistically significant for a base temperature of 18 °C (i.e. HDD_18_). For this base temperature, population-weighted heating degree-days have fallen at 1.5x the rate of area-weighted degree-days.

Across both area-weighted and population-weighted degree-days, choice of base temperature determines both the absolute value of the degree-day calculation and their rate of change over time. This is particularly pronounced in population-weighted degree-days, where for cooling, CDD_18_ have risen at 2.9x the rate for CDD_25_, and HDD_18_ have fallen at 2.0 the rate of HDD_10_.

Most importantly, this paper has shown that changes in population distribution between 1981 and 2018 heavily influence degree-days calculations. The global shift from a heating dominated regime to a cooling one is determined both by increasing global temperatures but also the relative growth of populations in warmer climates. From a sustainability and well-being standpoint, it is essential that these populations have access to zero-carbon buildings which provide comfortable environments.

## Funding

Malcolm N. Mistry was funded by a grant from the European Research Council (ERC) under the European Union's Horizon 2020 research and innovation programme, under grant agreement No. 756194 (ENERGYA).

Harry Kennard, Tadj Oreszczyn and Ian Hamilton acknowledge the following funding sources: CREDS (Engineering and Physical Sciences Research Council Centre for Research in Energy Demand Solutions (EP/R035288/1)), the Lancet Countdown (as an unrestricted grant from the Wellcome Trust (209734/Z/17/Z)) and SERL (Smart Energy Research Lab EP/P032761/1).

## Author contributions

All authors contributed the manuscript preparation and writing. TO, IH & HK conceived of the study. MM & HK conducted the data analysis.

## Declaration of Competing Interest

The authors declare that they have no known competing financial interests or personal relationships that could have appeared to influence the work reported in this paper.

## Data Availability

Data will be made available on request.

## Footnotes

1 It is assumed that the published article referenced here includes an error for which the base temperatures for the CDDs and HDDs were interchanged, which is corrected here.

## References

[1] Abergel T., Delmastro C. (2020).

[2] Taylor B.L. (1981). Population-weighted heating degree-days for Canada. Atmos. Ocean.

[3] ASHRAE. *2009 ASHRAE Handbook - Fundamentals (SI Edition)*. (American Society of Heating, Refrigerating and Air-Conditioning Engineers, 2009).

[4] Mistry M.N. (2019). Historical global gridded degree-days: A high-spatial resolution database of CDD and HDD. Geosci. Data J..

[5] Degree-Days C.I.B.S.E. (2006).

[6] Azevedo J.A., Chapman L., Muller C.L. (2015). Critique and suggested modifications of the degree days methodology to enable long-term electricity consumption assessments: a case study in Birmingham, UK. Meteorol. Appl..

[7] Kreider J.F., Curtiss P.S., Rabl A. (2009).

[8] Meng Q., Xi Y., Zhang X., Mourshed M., Hui Y. (2021). Evaluating multiple parameters dependency of base temperature for heating degree-days in building energy prediction. Build. Simul..

[9] IPCC (2021).

[10] Spinoni J., Vogt J.V., Barbosa P., Dosio A., McCormick N., Bigano A., Füssel H.-M. (2018). Changes of heating and cooling degree-days in Europe from 1981 to 2100. Int. J. Climatol..

[11] Harvey L.D. (2020). Using modified multiple heating-degree-day (HDD) and cooling-degree-day (CDD) indices to estimate building heating and cooling loads. Energy Build..

[12] Son J.-Y., Liu J.C., Bell M.L. (2019). Temperature-related mortality: a systematic review and investigation of effect modifiers. Environ. Res. Lett..

[13] Zhao Q. (2021). Global, regional, and national burden of mortality associated with non-optimal ambient temperatures from 2000 to 2019: a three-stage modelling study. Lancet Planet Health 5,.

[14] Nazarian N., Lee J.K.W. (2021). Personal assessment of urban heat exposure: a systematic review. Environ. Res. Lett..

[15] Huebner G.M., Chalabi Z., Hamilton I., Oreszczyn T. (2019). Determinants of winter indoor temperatures below the threshold for healthy living in England. Energy Build..

[16] Guttman N.B. (1983). Variability of population-weighted seasonal heating degree days. J. Appl. Meteorol. Climatol..

[17] Tol R.S.J. (2017). Population and trends in the global mean temperature. Atmósfera.

[18] Tuholske C. (2021). Global urban population exposure to extreme heat. Proc. Natl. Acad. Sci. USA.

[19] Atalla T., Gualdi S., Lanza A. (2018). A global degree days database for energy-related applications. Energy.

[20] Spinoni J., Barbosa P., Füssel H.-M., McCormick N., Vogt J.V., Dosio A. (2021). Global population-weighted degree-day projections for a combination of climate and socio-economic scenarios. Int. J. Climatol..

[21] Deroubaix A., Labuhn I., Camredon M., Gaubert B., Monerie P.-A., Popp M., Ramarohetra J., Ruprich-Robert Y., Silvers L.G., Siour G. (2021). Large uncertainties in trends of energy demand for heating and cooling under climate change. Nat. Commun..

[22] Biardeau L.T., Davis L.W., Gertler P., Wolfram C. (2020). Heat exposure and global air conditioning. Nat. Sustainability.

[23] IEA (2018).

[24] Mourshed M. (2012). Relationship between annual mean temperature and degree-days. Energy Build..

[25] Muñoz-Sabater J. (2021). ERA5-Land: a state-of-the-art global reanalysis dataset for land applications. Earth Syst. Sci. Data Discuss..

[26] SEDAC. Global Population Count Grid Time Series Estimates, v1 (1970 – 2000). (NASA Socioeconomic Data and Applications Center (SEDAC), Palisades, NY, 2011).

[27] SEDAC. Gridded Population of the World, Version 4 (GPWv4): Population Count, Revision 11. (NASA Socioeconomic Data and Applications Center (SEDAC), Palisades, NY, 2018).

[28] Vadodaria K., Loveday D.L., Haines V. (2014). Measured winter and spring-time indoor temperatures in UK homes over the period 1969–2010: A review and synthesis. Energy Policy.

[29] Humphreys M.A., Fergus Nicol J. (2018).

