# Supplementary material for: Population-weighted degree-days: The global shift between heating and cooling
Source: Energy Build. 2022 Sep 15;271:None. doi: 10.1016/j.enbuild.2022.112315 (PMC10502910; doi:10.1016/j.enbuild.2022.112315)
Supplement: Supplementary Data 1 [file mmc1.docx]

Supplementary analysis

1. Comparison between ERA5-Land population weighted degree-days and UK numbers.

Figure 9 shows HDDs for Great Britain derived by the UK government department of Business Energy and Industrial Strategy ^1^ and those derived from the ERA5-Land data used in this study. There is strong agreement between the two time-series for the years of common availability. Heating degree-days differ by a mean of 1.2 degree-days (0.03%) with a maximum difference of 58 degree-days (2.9%)


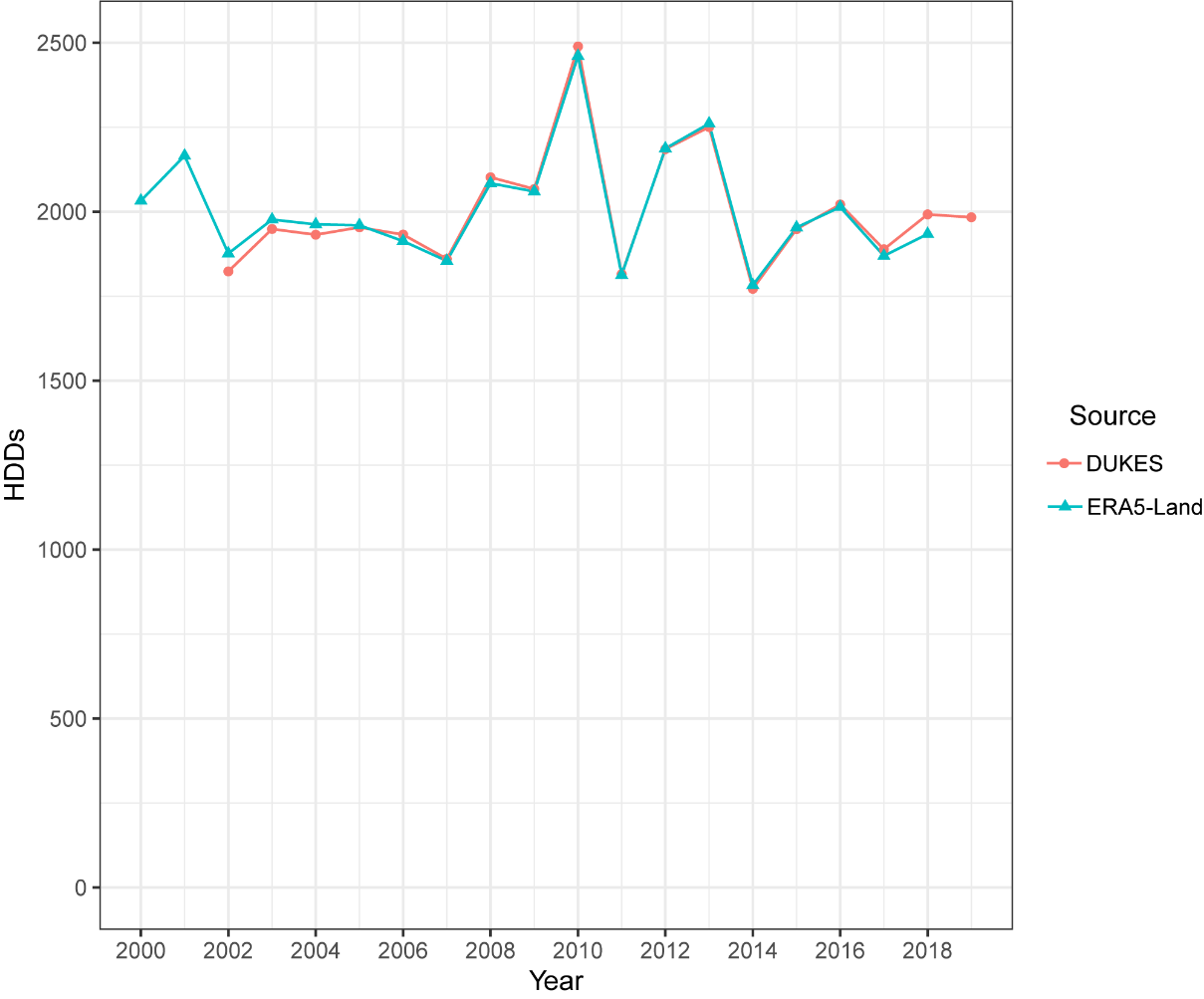


**Figure S1 Comparison between DUKES total heating degree-days for Great Britain and the those derived by this study**

1. Comparison between ERA5-Land population weighted degree-days vs EIA values.

Figure 10 shows ERA5 population weighted degree-days at a base temperature of 18.3°C (65°F) and those computed by the U.S. Energy Information Administration^2^. The two time-series agree less well than the UK example above. The average difference is 36.6 degree-days (4.73%) , with a maximum difference of 70.1 degree-days (8.31%). One possibility for this difference may be in part explained by differences in the population weighting method that the EIA uses, which employs state-wide population weightings and would therefore not be sensitive to within-state population heterogeneities ^3^.


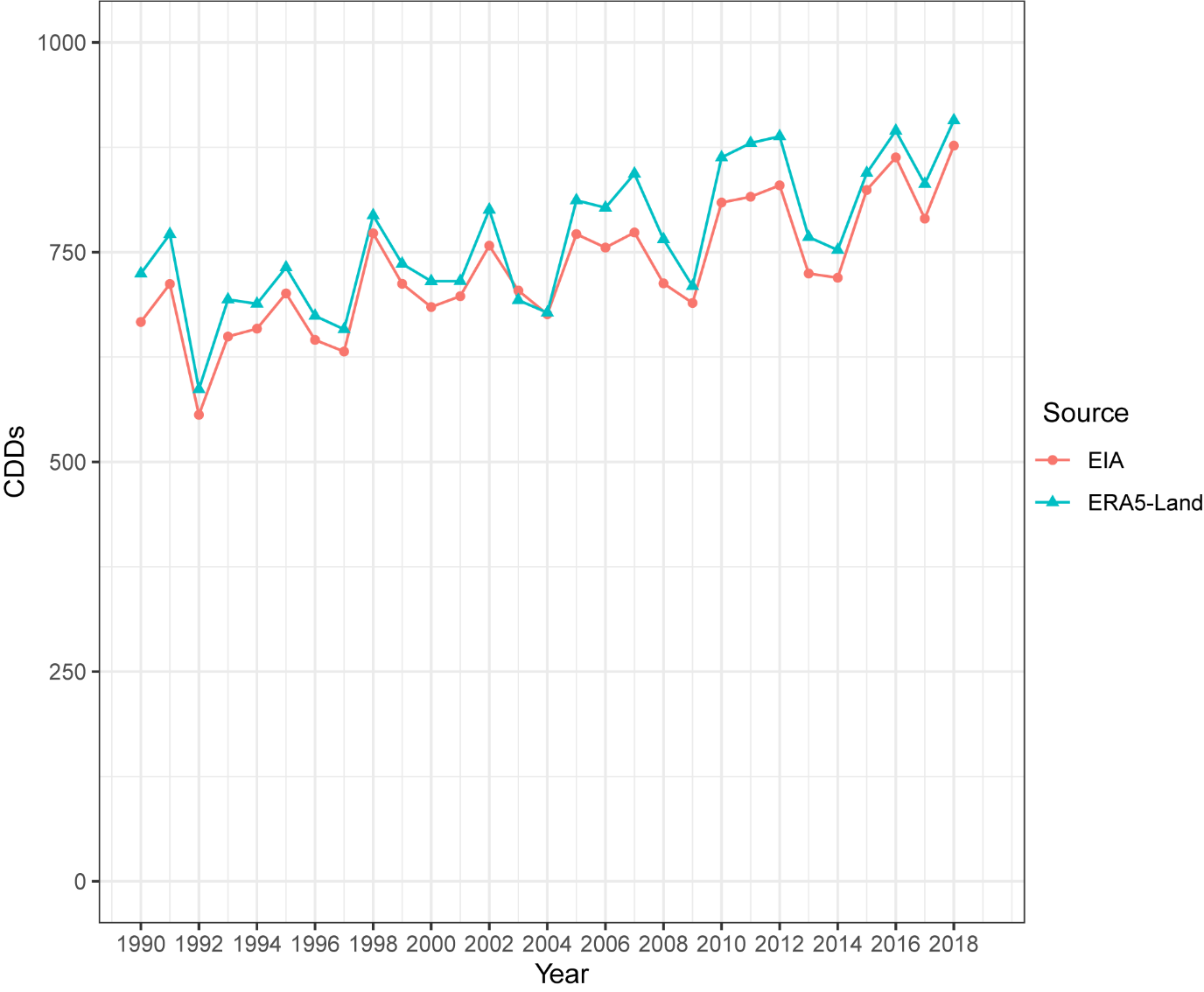


**Figure S2 Comparison of cooling degree-days at base temperature 18.3**°**C (65**°**F) calculated by EIA and those calculated by ERA5, the present study.**

## Intercept uncertainty

Estimating the uncertainty intervals on the intercept of two lines is not trivial. Here we use a graphical approach following Carter^4^, which over estimates the uncertainty in the intercept. Accordingly, the dark grey area where the two confidence regions overlap gives a conservative (overly wide) estimate of the uncertainty in their intercept. This allows us to arrive at the broad estimate of approximately ± 3 years for the intercept years given in the main text. Two examples are given below in figure S3.


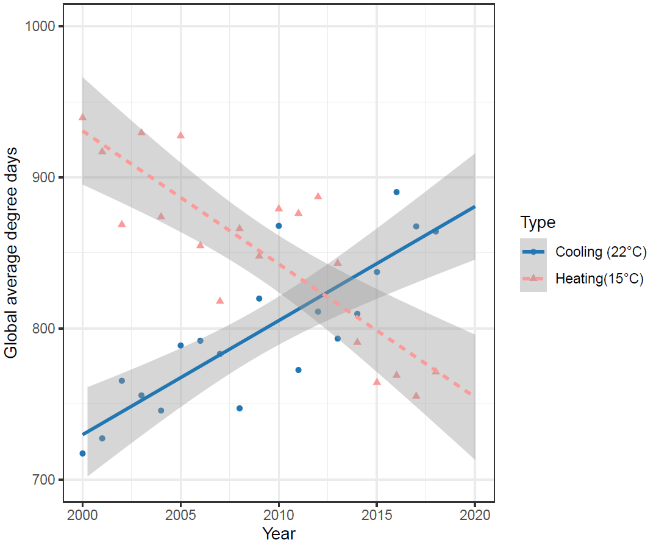

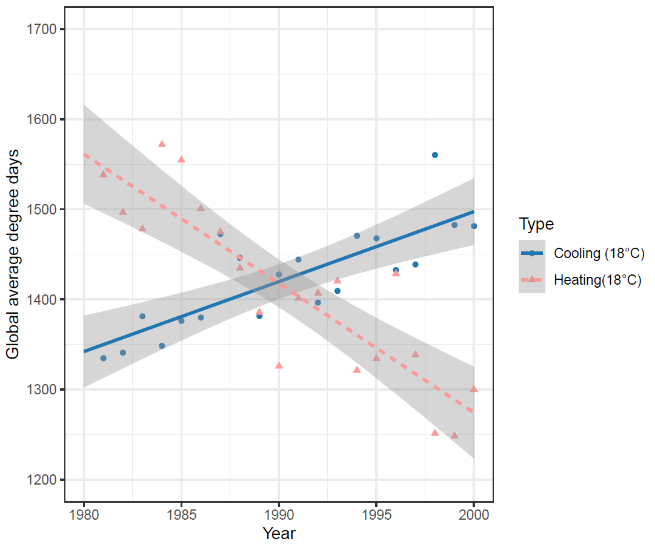


**Figure S3 Indication of uncertainty in the intercept between cooling and heating degree-days for two combinations of base temperature. Grey shaded areas indicate the 97.5% confidence intervals of each line, with the overlap therefore indicating the 95% confidence level of the intercept.**

## References

1 Harris, K. Digest of UK Energy Statistics. (Departement of Business, Energy and Industrial Strategy, London, 2020).

2 Energy Information Administration. Short-Term Energy Outlook Data Browser. (2021). <<https://www.eia.gov/outlooks/steo/data/browser/#/?v=28&f=A&s=&start=1997&end=2020&id=&map=&ctype=linechart&maptype=0&linechart=ZWCDPUS~ZWHDPUS>>.

3 Energy Information Administration. Short-Term Energy Outlook Supplement: Change in Regional and U.S. Degree-Day Calculations. (EIA, Washington DC, 2012).

4 Carter, K. N., Scott, D. M., Salmon, J. K. & Zarcone, G. S. Confidence limits for the abscissa of intersection of two least-squares lines such as linear segmented titration curves. *Anal. Chem.* **63**, 1270-1278, doi:10.1021/ac00013a017 (1991).
